# Proposal for improving access to palliative care by enhancing primary care services in an upper middle-income country

**DOI:** 10.3332/ecancer.2023.1514

**Published:** 2023-02-28

**Authors:** Malar Velli Segarmurthy, Richard Boon-Leong Lim, Salimah Othman, Sri Wahyu Taher, Harenthri Devy, Nirmala Bhoo-Pathy

**Affiliations:** 1 Centre for Epidemiology and Evidence-Based Practice, Department of Social and Preventive Medicine, Faculty of Medicine, Universiti Malaya, Kuala Lumpur 50603, Malaysia; 2 Training Division, Ministry of Health Malaysia, Precinct 3, Putrajaya 623675, Malaysia; 3 Department of Palliative Care, Hospital Selayang, Ministry of Health Malaysia, Kuala Lumpur 68100, Malaysia; 4 Family Health Development Division, Ministry of Health Malaysia, Putrajaya 62590, Malaysia; 5 Klinik Kesihatan Simpang Kuala, Ministry of Health Malaysia, Alor Setar 05400, Malaysia

**Keywords:** serious health-related suffering, palliative care, primary care, low- and middle-income

## Abstract

**Background:**

Primary care doctors play an important role in providing palliative care as they are often the first point of contact for most healthcare needs in the community. This mixed-method study aims to 1) determine the accessibility of palliative care services in Malaysia, an upper middle-income country with universal health coverage, 2) explore the knowledge, challenges and opportunities faced by primary care doctors in providing palliative care and 3) identify if minimum standards for palliative care service are clearly defined, available and achieved in primary care facilities.

**Methods:**

Data on availability of palliative care services will be sourced from governmental and non-governmental databases and reports. Accessibility will be examined by estimating the distance, travel time and cost to the nearest facility offering palliative care services from various locations throughout Malaysia. In-depth interviews will be conducted with primary care doctors to explore their knowledge, challenges and opportunities in providing palliative care. Alongside, a survey will be conducted to evaluate whether components of palliative care services are available in primary care facilities using the Minimum Standard Tool for Palliative Care from India, which covers all the domains recommended by the World Health Organization. All findings will be inductively analysed and integrated, followed by a strengths, weaknesses, opportunities and threats analysis and a threats, opportunities, weaknesses and strength analysis with relevant stakeholders.

**Expected results:**

The mapping study will provide empirical data on availability and accessibility of palliative care services in Malaysia. The qualitative inquiry will provide insights on the experiences and concerns of primary care physicians in providing palliative care in the community settings. The survey meanwhile will provide real-world data on availability of basic palliative care service components in the primary care facilities.

**Expected conclusion:**

Findings will facilitate development of framework and policies aiming to optimise provision of sustainable palliative care services at the primary care level in local settings.

## Background

Serious health-related suffering (SHS) will be the main cause of death for about 83% of the global population by 2060 [1]. Palliative care aims to provide holistic support to help both patients with SHS and also their caregivers to cope with the illness [2]. It ideally begins at the point of diagnosing a life-threatening or debilitating disease such as cancer and continues throughout disease trajectory till death and bereavement [3]

Malaysia is an upper middle-income country with universal health coverage where highly subsidised healthcare is provided to all citizens via a nationwide network of public hospitals and primary care clinics, irrespective of health insurance status [4]. The country is presently facing an increasing burden of non-communicable diseases resulting in greater need for palliative care [5]. Notably, palliative care services in Malaysia are predominantly based in urban areas, and mainly provided by palliative medicine physicians in inpatient settings, with only a handful of non-governmental organisations (NGOs) providing services in the community settings [5]. Furthermore, there are currently only 21 qualified palliative medicine physicians in the nation, most of whom are serving in the public sector. Although the number of specialists is set to grow, it would not be adequate to meet the growing needs of the population within the next decades [5].

Empowering the primary care doctors may therefore be a highly pragmatic approach as they are the gatekeepers of the health system, putting them in an ideal position to address the unmet palliative care needs of people with SHS [3]. A very good example of a working model for the above is from the state of Kerala in India. In 2008, the Government of Kerala integrated palliative care services into the primary care by establishing a cohesive, community-based palliative care delivery system, which includes training of lay volunteers. Kerala have since made remarkable achievements in provision of palliative care, as reflected by the fact that approximately 90% of palliative care service that is currently provided in India is now concentrated in this state [6]. Adopting a somewhat similar approach in Malaysia, where the primary care physician workforce from the public and private sectors is harnessed to not only provide palliative care services but also to train lay volunteers in providing basic services [7] may conceivably lead to substantial improvements in access and quality. Equally pertinent will be the need to gauge whether at the health systems level, the primary care clinics are able to cater for the medical needs of people requiring palliative care. Currently, there are no established minimum standards for primary care facilities to provide palliative care services in Malaysia.

To this end, a mixed-method study will be conducted to 1) determine the availability and accessibility of palliative care services in Malaysia, an upper middle-income country with universal health coverage, 2) explore the knowledge, challenges and opportunities faced by primary care doctors in providing palliative care in the community settings and 3) identify if minimum standards for palliative care service are clearly defined, available and achieved in primary care facilities in Malaysia.

## Method

The proposed research will utilise a convergent parallel approach, where three studies will be done concurrently, followed by data analysis of individual studies and finally integration and interpretation of the outcomes [8].

A geographical mapping study will be conducted to determine the availability and accessibility of palliative care services in Malaysia in terms of distance, travel time and cost of travel. Here, the comprehensiveness of data will be ensured by obtaining the most recently available data, which are updated on an annual basis by various stakeholders, namely from the Ministry of Health Malaysia and the Malaysian Hospice Council (MHC), which is the largest hospice NGO in Malaysia. Data on palliative care services that are provided at the hospital settings (public and private sectors) will be obtained from the national head of palliative care services in the Ministry of Health Malaysia. Data on palliative care service providers in the community settings will be retrieved from the latest records of the MHC given that the organisation has been regularly collecting data on independent, non-profitable community palliative care organisations in the nation. In addition, data will also be obtained from the formal records of the Family Health Development Division of Ministry of Health Malaysia, which is in charge of the domiciliary care services provided by the government health clinics. This database was set up in 2014.

The route planner under ‘Get Directions’ in Google Maps will be used to determine the distance from each district in Malaysia to the closest palliative care unit or clinic offering palliative care and domiciliary care, or the closest community-based palliative care service. The points located at the geographical centre of the districts will be selected as the starting point and the nearest palliative care centre’s main entrance as the end points. Travel time and the cost of travel will be estimated via Google Maps and supplemented by ‘Rome2rio’, a web-based application that provides information on the availability of public transportation as well as the time taken to travel and the estimated cost of travelling from one point to another [9]. The estimated cost of travel will be calculated on the same day to avoid inflationary changes in the currency. In instances where land travel is possible but public transportation is not available, travel costs will be calculated based on the distance that needs to be covered (USD 0.16 per km)

A qualitative study will be undertaken to explore the knowledge, challenges and opportunities faced by primary care doctors in providing palliative care services in the Malaysian setting, whereby in-depth interviews (IDIs) will be conducted via telephone calls or virtual meetings. Primary care doctors with a valid annual practicing certificate will be eligible for this study. Using the national register of primary care doctors obtained from the Medical Practice Division of the Ministry of Health Malaysia, primary care doctors from private practices, as well as from ‘*Klinik Kesihatan’* (public primary care clinics) will be sampled (stratified by age, state and location of practice (urban versus rural)). An interview guide comprising open-ended, nondirective questions will be developed based on literature review and expert opinions. It will cover three main domains, namely knowledge, challenges and opportunities faced by primary care physicians in providing palliative care to their patients. The interviews will be recorded with permission and supplemented with notes taken by the researcher. It is expected that data saturation will be achieved after about 15 to 20 IDIs [10].

The online survey will utilise a standard audit tool, which aims to evaluate the ‘essential’ and ‘desirable’ components of palliative care services in primary care settings. Here, the Minimum National Standards for Palliative Care for India, which is a self-evaluation tool for organisations providing palliative care will be utilised [11].

Notably, the domains that are explored in the Indian audit tool for palliative care programmes are in line with the recommendations of the World Health Organization (WHO) on planning and implementing palliative care services [12]. WHO has emphasised that these domains of care are crucial to ensure that palliative care and basic pain management are gradually established, especially in low-resource situations [11], encompassing process and structure of care, drug availability, training of personnel, quality, organisational aspects as well as the legal and ethical aspects. The tool also covers psychosocial, physical and spiritual aspects [7]. Prior to its use, an expert panel comprising public health professionals, palliative medicine physicians, policy makers and family medicine specialists will be convened to go through the standard audit tool to adapt it for local use. Following this, 400 primary care facilities will be sampled from the list of primary care facilities obtained from the Health Informatics Centre of Ministry of Health Malaysia via a stratified sampling method to ensure that there is adequate representation of urban and rural clinics, as well as private and public facilities from the whole country. Sample size was calculated using the formula recommended by Aday and Cornelius [13]. Upon filling up the informed consent, the clinical head of the primary care clinics will be asked to fill up the standard audit tool on behalf of the respective facility.

In this mixed-method study, both the inductive and deductive (hybrid) approaches will be used to thematically analyse data. The qualitative research will follow inductive logic, where we will begin with no codes and then develop the codes as the transcribed dataset is analysed. Findings from the mapping study will also be inductively analysed to identify gaps in availability and accessibility of palliative care services in Malaysian districts. As for the survey, we will analyse findings based on the responses of primary care doctors on whether the minimum standards of basic palliative care services are available in the primary care clinics across various states in Malaysia. Findings from all three studies will be categorised under domains such as experiences, challenges, knowledge, structure and processes of care, training of personnel, physical, psychosocial and spiritual dimensions, ethical and legal aspects, organisational aspects, quality and drug availability. The stakeholders engagement is expected to deductively analyse and identify existing gaps [14].

A face-to-face stakeholder engagement will be conducted to identify strengths, weaknesses, opportunities and threats analysis of each identified domain. The stakeholders will comprise palliative medicine physicians, public health physicians, family medicine specialists, general practitioners from public and private sectors, health policy makers, NGOs providing palliative care and patient support group representatives. This will be followed by threats, opportunities, weaknesses and strength analysis to examine external opportunities and threats and comparing them to strengths and weaknesses, against internal opportunities for each individual domain.

Table 1 highlights the characteristics and various domains addressed by all three studies.

### Statistical analysis plan

Data for the mapping study, and survey of primary care clinics will be analysed using the Statistical Package for Social Sciences version 23 [15].

For the mapping study, the median distance, travel time and costs to the nearest facility offering palliative care services will be determined. As for the survey, a descriptive analysis will be carried out to determine the distribution of primary care clinics (type of facility, size of facility, position of the respondent). Thereafter, mean score will be calculated by summing up the points for each question in the standard tool and dividing the sum by the total number of respondents.

Transcribed data from the IDIs will be managed using NVivo 2, a computer-assisted qualitative data analysis software. Coding reliability approach will be used to thematically analyse the data [16]. Data analysis will be done by systematically searching, reading and arranging the interview transcripts and notes. Textual data will be divided into segments, examining the similarities and differences. Based on these segments, broad themes and codes will be allocated. The identified segments will be grouped with conceptually similar data in their respective nodes. If a category or code does not fit into the established matrix, a separate category or code will be developed. This is to ensure that all data are captured, irrespective of whether they fitted into the existing model or otherwise. Each of the theme and subtheme will then be explained in detail alongside the excerpts from the interview transcripts [17, 18].

Once data from all three studies are obtained, they will be compared, integrated and interpreted to provide a broader overview.

### Ethical consideration

Ethical approvals have been obtained from the Medical Review & Ethics Committee of the Ministry of Health Malaysia. Informed consent for the qualitative study will be obtained electronically from the study participants. For the survey, approvals will also be obtained at the institutional level prior to getting the clinical administrators in the respective clinics to respond to the standard audit tool. Permission to use/adopt the Minimum Standard Tool for Palliative Care in India has also been obtained.

## Discussion

The provision of palliative care in Malaysia is currently fragmented where patients and caregivers rely heavily on support from tertiary care. In an ideal setting, the population who needs it, who are the sickest and most vulnerable should be able to obtain high-quality palliative care service from all layers of healthcare facilities. Incorporating palliative care into health services at the primary care level as part of universal health coverage is a great way of achieving this [11].

Prior evidence has demonstrated that hospital costs were significantly lower for patients who received palliative care consultation compared to those who did not [18]. Particularly, patients who received community-based palliative care reported a 20% reduction in total medical costs ($619 per enrolled member per month), which was also accompanied by a 33% reduction in hospital admissions, 38% reduction in intensive care unit admissions and 12% reduction in total hospital days throughout their disease trajectory [19].

The findings from the palliative care mapping study will provide empirical evidence on accessibility to palliative care services in the Malaysian setting. With only about 10% of the population who are in need of palliative care receiving it currently in Malaysia, it is important to take urgent actions to improve access to palliative care services [5]. Globally, there is a growing interest in provision of palliative care in the community settings or at home to better meet the needs of a growing population of patients who are dealing with SHS [20]. Notably, the primary care doctors are well-positioned to provide palliative care as they are the closest to the community and often the first point of contact for most healthcare needs. Care closer to home can help in improving the comfort level and reducing travel costs for patients with SHS [21]. Furthermore, it will also reduce caregiver exhaustion where much time is not wasted on travelling, or waiting for consultations in tertiary hospitals [21]. Palliative care provision at the primary care level is also efficient for health systems as it can help reduce recurrent and unnecessary hospitalisations.

In order to decentralise palliative care, the primary care doctors must be given adequate support to cater to the needs of the population. The qualitative study will focus on the challenges faced by primary care doctors as well as the opportunities that can be utilised to enhance palliative care service delivery in primary care settings. Although Malaysia has developed a national strategic plan for palliative care services, there are many unheard challenges and barriers that need to be highlighted to the policymakers to be tackled to meet the growing needs. Thus, findings from the qualitative inquiries is expected to provide in-depth understanding of the experiences and concerns of primary care physicians, which in turn will be useful in development of strategies to empower the primary care workforce with knowledge and skills to provide palliative care at the community level.

Apart from improving accessibility and empowering primary care doctors, it is imperative to ensure that the health system is adequately supporting the delivery of high-quality palliative care service at the primary care level. Since palliative care service requires a team approach, the team needs a structured direction as well as resources to drive them to deliver the service. To this end, findings from the survey will enable us to gauge the extent to which minimum standards to provide basic palliative care is met in primary care facilities across the nation. This in turn will enable development of actionable solutions at the provider’s level to ensure that existing issues at the primary care facilities are addressed and medical resources (e.g. drug availability) are adequate to cater to the needs of the population with SHS.

It is acknowledged that this mixed-method study will suffer from several limitations. For instance, in the current study, accessibility will only be gauged using simple measures of distance, travel time and cost. Future studies, for instance, could employ more advanced statistical approaches to highlight inequities in access to palliative care services including the use of Gini Index, or spatial analytical techniques.

Furthermore, the mapping study will only capture registered centres, which formally offer palliative care services. Hence, informal providers of palliative care in the nation may be missed. Also, it is anticipated that we may be underestimating the actual distance, travel time and travel costs to the nearest centres offering palliative care in rural regions such as in East Malaysia, where land transport is lacking. Regardless, our findings may still serve as a conservative estimate. Furthermore, the qualitative study will include a relatively small sample of primary care physicians who may not be entirely representative of the primary care workforce in Malaysia. Meanwhile, in the third study, although we plan to randomly sample clinics from the urban and rural locations, as well as public and private settings, response rates may not necessarily be uniform due to factors such as lack of time or motivation.

## Conclusion

The existing network of palliative care services in Malaysia has to be strengthened and widened so that all healthcare professionals at all layers of health facilities will be trained and credentialled to provide basic palliative care to the population [22]. To this end, data gathered from this study as well as the strategies derived from the mentioned analysis can be used as an evidence base to develop solutions to decentralise palliative care in Malaysia. Specifically, evidence from the study can facilitate in empowering primary care physicians, as well as in improving availability of essential medicines for pain relief and symptom control in the primary care settings. Ultimately, study findings will be useful in improving access to palliative care services at the community level in Malaysia, which in turn will lead to improved quality of life and reduced suffering for patients and families dealing with SHS.

## Author contributions

Conceptualisation: Nirmala Bhoo-Pathy, Malar Velli Segarmurthy, Richard Lim Boon Leong

Methodology: Nirmala Bhoo-Pathy, Malar Velli Segarmurthy

Original draft: Nirmala Bhoo-Pathy, Malar Velli Segarmurthy

Writing – review & editing: Nirmala Bhoo-Pathy, Malar Velli Segarmurthy, Salimah Othman, Sri Wahyu Taher, Harentri Devy

Final approval: Nirmala Bhoo-Pathy, Malar Velli Segarmurthy, Salimah Othman, Sri Wahyu Taher, Harenthri.

## Conflicts of interest

All authors declare that there are no potential conflicts of interest.

## Funding

This research did not receive any specific grant from funding agencies in the public, commercial or not-for-profit sectors.

## Figures and Tables

**Table 1.** Characteristics and issues addressed by the mapping study, IDI and online survey.

Mixed-method study			
**Type of study**	**IDI**	**Online survey** (Adopted from the standard audit tool for Indian palliative care programme)	**Mapping study**
Unit of analysis	Primary care doctors	Primary care facilities	Palliative care services at various levels
Domains explored	Knowledge Challenges Opportunities	Structure and processes of care Training of personnel Physical, psychosocial and spiritual dimensions Ethical and legal aspects Organisational aspects Quality Drug availability	Types of services Distance, travel time and costs to the nearest center offering palliative care service
Sample size	20 primary care doctors	400 primary care facilities	124 facilities offering palliative care service in all 104 districts in Malaysia
